# Differential effects of cannabis constituents on schizophrenia-related psychosis: a rationale for incorporating cannabidiol into a schizophrenia therapeutic regimen

**DOI:** 10.3389/fpsyt.2024.1386263

**Published:** 2024-04-23

**Authors:** Kennadi Johnson, Abby J. Weldon, Melissa A. Burmeister

**Affiliations:** ^1^ William Carey University School of Pharmacy, Departments of Pharmaceutical Sciences and Pharmacy Practice, Biloxi, MS, United States; ^2^ William Carey University School of Pharmacy, Department of Pharmaceutical Sciences, Biloxi, MS, United States

**Keywords:** schizoprenia, cannabidiol (CBD), delta-9-tetrahydrocannabinol, psychoses, cannabis

## Abstract

Schizophrenia is a serious mental health disorder that confers one of the highest mortality rates of all psychiatric illnesses. Although the disorder’s psychotic symptoms are treatable with conventional antipsychotics, they remain incurable. Moreover, medication adherence is poor, and individuals with schizophrenia choose to self-medicate with illicit substances, including cannabis. It is well-established that the delta-9-tetrahydrocannabinol (delta-9-THC) component of cannabis elicits psychotomimetic effects at high doses; worsens schizophrenia-related psychosis; commonly develops into cannabis use disorder in individuals with schizophrenia; and increases the risk of earlier-onset schizophrenia symptoms in those harboring genetic susceptibility. However, individuals with schizophrenia commonly use cannabis and cannabis derivatives such as cannabidiol (CBD). These products seem to alleviate psychotic symptoms and relieve adverse side effects of antipsychotic medications. Therefore, one notion that has gained traction is the potential utility of cannabis-derived cannabidiol (CBD) as adjunct treatment to reduce schizophrenia-associated psychosis and other symptoms. Currently, preclinical and clinical data remain inconclusive. The present review distinguishes the mechanisms underlying schizophrenia-associated vs. cannabis-induced psychosis; reviews the evidence for delta-9-THC-mediated exacerbation vs. CBD-mediated amelioration of schizophrenia-associated psychosis; and describes potential approaches for incorporating CBD into schizophrenia therapeutic regimen in a safe and efficacious manner.

## Introduction

Schizophrenia is a chronic, severe, and debilitating mental health disorder that affects ~1% of adults globally, including ~1.2% of the U.S. population (1, 2). The word “schizophrenia” encompasses two phenomena: “schizo-” meaning splitting and “-phrenia” meaning mind. The disorder is clinically heterogeneous, with symptoms categorized as positive, negative, and cognitive (3). Positive symptoms include auditory and visual hallucinations, delusions, suspiciousness, and abnormal motor behavior (3). The five cardinal negative symptoms are blunted affect (*i.e.*, impaired ability to express emotions), alogia (*i.e.*, fewer spoken words), asociality (*i.e.*, aversion to social interaction and/or preference for isolation), anhedonia (*i.e.*, inability to feel pleasure), and avolition (*i.e.*, total lack of motivation) (3). Cognitive symptoms are less specific and include disorganized attention, speech, and/or thought, which could impede communication (3). Schizophrenia symptoms often manifest between late adolescence and early adulthood, with a gender-specific timing (4). Men are more likely to be diagnosed with schizophrenia at an earlier age than women, suggestive that estrogen is protective (4). Males are commonly diagnosed between late adolescence and their early twenties, whereas females are oftentimes diagnosed between their twenties and thirties (4). Ethnicity also plays a role in the prevalence of schizophrenia. Risk is substantially greater in individuals of Afro-African and Afro-Carribean descent, partly due to poor social structure (*e.g.*, structural racism, lower socioeconomic position, history of migration) (5, 6). These groups are also most commonly misdiagnosed (6, 7).

Approximately half of individuals with schizophrenia have psychiatric comorbidities. Depression occurs in 23-57% of affected individuals (8). Anxiety disorders including panic disorder, posttraumatic stress disorder, obsessive-compulsive disorder, generalized anxiety disorder, and social anxiety disorder are also common (8). Cardiometabolic disturbances (*e.g.*, heart disease, liver disease including hepatic steatosis, insulin resistance, type 2 diabetes mellitus) frequently accompany schizophrenia independent of second-generation antipsychotic medication use, with many individuals succumbing to cardiovascular disease (9–11). Schizophrenia is underdiagnosed; consequently, it is difficult to precisely quantify disease duration (12). Individuals with schizophrenia have a heightened risk of premature mortality, with death estimates of 15-30 years before healthy individuals (12, 13). Premature mortality may be partly attributable to under-detection, inadequate treatment, unhealthy lifestyle, and/or comorbidities (12, 13). Nearly 5% of people with schizophrenia commit suicide (12). Collectively, schizophrenia has one of the highest mortality rates amongst all psychiatric disorders (13).

Of all schizophrenia’s psychiatric comorbidities, substance use disorder dominates (8, 14, 15). Alcohol, tobacco, and cannabis are commonly abused. Although the precise reasons why individuals resort to illicit drug use remain unknown, multiple hypotheses exist. The “self-medication” hypothesis suggests that individuals with schizophrenia use drugs and alcohol to relieve adverse psychiatric manifestations and/or reduce neurological side effects (*e.g.*, restlessness, parkinsonism) caused by antipsychotic medications (16). Another hypothesis suggests that dysregulation of the mesocorticolimbic dopamine pathway not only contributes to the development of schizophrenia but also leads to “reward deficiency syndrome,” whereby illicit substances and alcohol are increasingly sought (15). Due to increased legalization of recreational and medicinal cannabis, it is used now more than ever before (17). Cannabis itself is implicated in the development of psychosis, whereby adult users are substantially more susceptible, particularly young males (18, 19). Adolescents (*i.e.*, 12-18 years of age) who use cannabis at low- or high-frequency display significantly increased risk for depression, anxiety, suicidality, and schizophrenia during early adulthood (20, 21).

Growing evidence indicates that the two primary constituents of cannabis, delta-9-tetrahydrocannabinol (delta-9-THC) and cannabidiol (CBD), differentially affect schizophrenia-related psychosis. Delta-9-THC exacerbates schizophrenia symptoms, whereas CBD alleviates them. Along with antipsychotic effects, CBD anecdotally decreases symptoms of neuroleptic-induced parkinsonism and exerts anxiolytic and anticonvulsive actions (22–25). A systematic review by Ahmed et al. examined the effects of delta-9-THC and CBD on schizophrenia symptoms from eleven clinical trials conducted from 2005-2021 (26). The authors concluded there was insufficient evidence to recommend medical cannabis to treat schizophrenia (26). However, the notion continues to gain traction, with several ongoing clinical trials (27–29). Here, we compare the etiopathophysiology underlying schizophrenia- vs. cannabis-related psychoses and revisit the rationale for adding CBD to conventional schizophrenia treatment regimens.

## Schizophrenia-related psychosis: etiopathophysiology and conventional treatments

The etiopathophysiology of schizophrenia is thought to involve genetic alterations coupled with structural brain changes and environmental factors. Genetic predisposition is a leading causal factor. An individual with a family history of schizophrenia has a significantly increased risk of developing the disorder, especially if both parents have the condition (30). Genetic polymorphisms and abnormalities in the growth factor neuregulin; the regulator of synaptic vesicle transport and neurotransmitter receptor trafficking dystrobrevin-binding protein 1; and the catecholamine o-methyltransferase are implicated (30, 31). Genome-wide associations studies have identified risk variants that increase susceptibility. These variants include copy number variants (CNVs) that are enriched in CNS inhibitory and excitatory neurons as well as in genes that encode for proteins governing multiple aspects of neuronal function, such as structure, function, maturation, and synaptic development (32–36). Since CNVs confer low fecundity, they must frequently occur as recurrent *de novo* mutations (34).

Anatomical and functional dysconnectivity between the prefrontal cortex and other brain regions (*i.e.*, the “dysconnectivity hypothesis”) contributes to the cognitive and behavioral impairments associated with schizophrenia (30, 37). Notable schizophrenia-related reductions in gray matter volume in the temporal and parietal lobes have been observed (30). Widespread and variable involvement of multiple brain regions and circuits reflect the complex genetic variation that underpins schizophrenia, with impaired dopaminergic neurotransmission heavily implicated (38, 39). Environmental factors include low birth weight, gestational diabetes, delivery via emergency Cesarean section (suggesting a role for gut microbiome dysbiosis), and vitamin D deficiency (30, 40, 41). Urban residence and wintertime birth increase the likelihood of developing the disorder by 2-4% and 10%, respectively (30). Childhood trauma and social isolation are psychological factors that may also increase risk (30).

The American Psychiatric Association recommends non-pharmacological therapy in conjunction with antipsychotic medication usage (42). Non-pharmacological interventions include cognitive-behavioral therapy and other psychosocial treatments, psychoeducation, and supported employment services (42). Commonly prescribed antipsychotic medications for first-time schizophrenia episodes include aripiprazole (Abilify), lurasidone (Latuda), quetiapine (Seroquel), and brexpiprazole (Rexulti) (42). Clozapine (Clozaril, FazaClo, Versacloz) is recommended to treat resistant schizophrenia (42). Antipsychotic medication side effects include sedation, orthostatic hypotension, dry mouth, constipation, and weight gain (42). Extrapyramidal effects such as akathisia (*i.e.*, restlessness with a strong, uncontrollable desire to move) and parkinsonism are more common with first-generation antipsychotic agents, manifesting weeks following treatment initiation or with dose increases (42). Antipsychotic medications, particularly second-generation agents, also increase the risk of metabolic syndrome and atherosclerotic cardiovascular disease (43, 44). Given these adverse effects, poor medication adherence is a major treatment barrier, with an estimated mean adherence rate of only 50% (45). Non-adherence is associated with poor outcomes, such as heightened risk of relapse, rehospitalization, suicidal ideations, aggressive behaviors, and mortality (42). These challenges warrant a better understanding of how adjunct therapies such as CBD could reduce adverse side effects as well as improve treatment outcomes.

## Adverse effects of cannabis on mental health

Cannabis is an illicit, psychoactive drug used by adolescents and adults (46). Legalization of cannabis for medicinal (currently 38 states) and recreational (currently 23 states) purposes is rising (47). Limited evidence indicates that recreational cannabis legalization in the U.S. increases the odds of transition to cannabis use amongst youths and adults (17, 47). The primary constituents of cannabis are delta-9-THC, CBD, delta-8-THC, and cannabinol (46). Delta-9-THC, the component that elicits psychoactive effects, exerts partial agonism at cannabinoid type 1 (CB1) and type 2 (CB2) receptors. CB1 receptors are located in brain regions that play critical roles in cognition, memory, emotion, movement, and attention, including the hippocampus as well as the frontal and temporal lobes (46, 48). In contrast, CB2 receptors are expressed in immune cells (46, 48). Receptor binding increases dopamine and glutamate release and inhibits GABA release (46, 48). Hyperactivity in dopaminergic, serotonergic, and alpha-adrenergic signaling, in conjunction with hypoactivity in glutaminergic and gamma-aminobutyric acid (GABA)ergic signaling, are well-implicated in the psychiatric manifestations of schizophrenia (30, 49). Acute intravenous (IV) administration of delta-9-THC to healthy individuals increases striatal glutamate and glutamine metabolite levels (collectively referred to as Glx) vs. placebo (50). Furthermore, baseline Glx impacts the magnitude of this response (50). Individuals with lower baseline Glx are more sensitive to the psychotomimetic effects of IV delta-9-THC (50). Also, previous cannabis use positively correlates with delta-9-THC-induced striatal Glx (50).

Health benefits of acute cannabis use include relief of pain, anxiety, stress, depression, and nausea (48). However, cannabis also induces adverse cognitive, emotional, and reward circuit deficits (48). In individuals who possess genetic susceptibility, regular cannabis use during their lifetimes increases their risk of developing schizophrenia, and cannabis-induced psychosis may progress to full-blown schizophrenia in these individuals (51). Long-term cannabis use structurally alters the brain, as revealed by magnetic resonance imaging (48). Chronic cannabis users display reduced gray and white matter volumes, including in the hippocampus, compared to nonusers (48, 52). Preclinical data from rat studies indicate that delta-9-THC use may lead to heritable molecular impairments, whereby an increased susceptibility towards developing psychiatric diseases is conferred to offspring (53). Germline exposure to delta-9-THC (via parental exposure) increases the work effort of F1 progeny (unexposed to the agent themselves) to self-administer heroin and enhances stereotyped acute withdrawal behaviors (53). Exposure to delta-9-THC also variably disturbs gene transcript and protein levels for cannabinoid, glutamatergic (both AMPA and NMDA), and dopaminergic receptors in the nucleus accumbens, dorsal and ventral striatum during both adolescence and adulthood (53). Aberrant signaling at these receptors impacts key components of the neural circuity that mediate reward sensitivity and compulsive behaviors. Long-term depression (*i.e.*, decreased postsynaptic strength) in the dorsolateral striatum of F1 offspring subjected to parental germline THC exposure is also increased (53).

## Delta-9-THC-mediated exacerbation vs. CBD-mediated alleviation of schizophrenia-related psychosis

Cannabis potency is dictated by the concentration of delta-9-THC (54, 55). Concomitant increases in delta-9-THC concentration and decreases in CBD concentration have contributed to an overall increased potency of multiple cannabis strains (56). However, the psychotomimetic property of cannabis reflects the delta-9-THC : CBD ratio (55, 57). Cases of cannabis use disorder (CUD) and reports of increased incidence of psychiatric illnesses in individuals who abuse cannabis have both risen (54). Although the precise mental health effects that delta-9-THC elicits in people with schizophrenia remains limited, data are emerging. Individuals with schizophrenia are more susceptible to the psychosis-inducing effects of delta-9-THC compared to healthy individuals (58). A randomized controlled trial including participants with schizophrenia receiving antipsychotic medication determined that delta-9-THC exacerbates positive and negative symptoms; worsens verbal learning and recall; and induces feelings of panic and tiredness (58).

Delta-9-THC worsens positive symptoms associated with schizophrenia, but CBD may mitigate them (55, 58, 59). In contrast to delta-9-THC, CBD lacks psychoactive activity and reward properties due to lack of agonism at CB1 and CB2 receptors. CBD has low binding affinity for these receptors with limited activity, but it displays antagonist activity in the presence of delta-9-THC (60). CBD behaves as a non-competitive negative allosteric modulator of CB1 and CB2 receptors, whereby it reduces the efficacy and potency of delta-9-THC (61–63). CBD also inhibits rodent fatty acid amide hydrolase (FAAH), resulting in increased levels of anandamide, which is associated with decreased clinical symptoms of schizophrenia (64, 65). Anandamide is an endogenous ligand of CB1 receptors, where it mediates signaling that blocks pain pathways and improves mood and congition (64). Preclinical rodent data indicate that elevated anandamide levels are associated with reduced psychotic-like behavior (66). Although high-dose CBD increases anandamide levels in CUD patients, it does not appear to do so by inhibiting human FAAH (67, 68). Additionally, activation of G protein-coupled receptor (GPR) 55 by delta-9-THC in dorsal root ganglion sensory neurons results in neuronal excitability, while CBD exerts opposite effects via antagonism at GPR55, contributing to its antipsychotic effect (69, 70). CBD also buffers the psychoactive effects of delta-9-THC by serving as an agonist at serotonin 5-HT1A receptors and transient receptor potential cation channel subfamily V member 1 (TRPV1) (71, 72).

In healthy individuals, CBD (600 mg) attenuates the psychotic-like behavior exacerbated by delta-9-THC (10 mg) (73). In individuals with schizophrenia, CBD (800 mg/day) is as effective as an equivalent dose of the atypical, second-generation, antipsychotic drug amisulpride, a dopamine (D2) receptor antagonist, in reducing positive and negative symptoms with fewer side effects (64). Co-administration of an antipsychotic agent and CBD (1,000 mg/day) to individuals with schizophrenia reduces positive symptoms to a greater extent compared to counterparts co-administered an antipsychotic medication and placebo (74). However, CBD (vs. placebo) does not improve schizophrenia-related cognitive impairment in patients diagnosed with chronic schizophrenia receiving antipsychotic treatment, and there is increased sedation (75).

Recent work by van Boxel et al. sheds light on potential mechanisms underlying CBD-mediated amelioration of psychosis. In patients diagnosed with either schizophrenia or a related psychotic disorder within five years and dosed with a stable antipsychotic agent for one month prior to study inclusion, adjunctive CBD therapy (600 mg/day) for twenty-eight days improved functional connectivity in the Default Mode Network (DMN) of the brain (76). The DMN becomes activated when individuals focus on internal mental-state processes, such as self-referential processing, interoception, autobiographical memory retrieval, and imagining the future. Early-onset psychosis patients generally display increased levels of glutamate metabolites and reduced concentrations of GABA, with conventional antipsychotic agents lowering brain glutamatergic metabolites (77–80). Prefrontal cortex levels of *N*-acetyl-aspartate (NAA), a biomarker of neuronal viability, are reduced in schizophrenia (81, 82). Prefrontal NAA levels positively correlate to glutamatergic metabolite concentrations only in unmedicated psychosis patients (vs. medicated patients or healthy controls), whereas prefrontal NAA levels correlate to GABA levels in medicated and unmedicated patients but not controls (83). van Boxel et al. observed that, whereas increased prefrontal NAA levels correlated with increased glutamate and GABA levels in placebo-treated study participants, they did not in CBD-treated counterparts (76). Instead, NAA levels negatively correlated to GABA and positively correlated (albeit marginally) to glutamate levels with CBD treatment (76). There was also decreased positive symptom severity, which was associated with reduced concentrations of glutamate and NAA in CBD-treated participants (76). Plasma THC concentrations were higher in placebo- vs. CBD-treated individuals, suggesting that CBD treatment may beneficially impact cannabis use and supporting previous findings that CBD effectively treats CUD (84). Also, CBD therapy did not alter brain activity during reward anticipation or receipt (76).

## Incorporation of CBD into a schizophrenia therapeutic regimen

Emerging evidence indicates the potential for administering CBD in conjunction with conventional antipsychotic medications (rather than as a standalone treatment) to curtail schizophrenia-related psychosis. The relative ease and practicality of incorporating CBD into antipsychotic therapeutic regimens stems, in part, from the fact that products infused with hemp- (vs. cannabis-) derived CBD (containing ≤0.3% THC) are now largely available for consumer purchase (85). However, there are challenges to supplementing schizophrenia medications with CBD. Dose-dependent side effects of CBD include hepatic abnormalities, diarrhea, fatigue, vomiting, and somnolence (85, 86). Cytochrome P-450 (CYP450) enzymes, particularly the CYP2C9, CYP2C19, and CYP3A4 isoforms, metabolize CBD to its primary active metabolite 7-hydroxy-CBD (85, 87–89). Serum CBD levels that are pharmaceutically active could become elevated when CBD is taken concomitantly with medications that inhibit these three enzyme isoforms (88, 90). Moreover, reduced CBD bioavailability could occur with medications that induce these isoforms (90). CBD inhibits CYP2C9, CYP2C19, and CYP2D6, and may inhibit members of the CYP3 family; alternatively, repetitive administration may induce members of the CYP2B family (90–92). CBD may antagonize the psychoactive effects of delta-9-THC partly by inhibiting CYP2C9, the primary isoenzyme that metabolizes delta-9-THC to more active metabolites (63). CBD-mediated alterations in drug metabolism create the potential for pharmacological interactions with multiple medication classes, including antiepileptics, antidepressants, opioid as well as non-opioid analgesics, CNS depressant drugs, immunosuppressants, and sympathomimetic agents (88, 90).

Regarding the use of CBD as adjunct therapy in a schizophrenia treatment regimen, many drug-drug interactions involving first- and second-generation antipsychotics occur at CYP3A4 and CYP2D6 (93). Consequently, co-administration of antipsychotic treatments and CBD could be problematic because they each serve as potential perpetrator drugs (*i.e.*, inhibitors) towards one another, whereby competitive (*i.e.*, reversible) inhibition ensues, which could increase the risk of adverse effects (85, 94). The degree of competitive inhibition is governed by the enzyme affinities and substrate concentrations (94). Alternatively, co-administration could reduce the amount of active metabolites generated by antipsychotic metabolism (95). It is generally recommended that substrates sharing the same CYP450 isoenzyme not be co-administered (85). Furthermore, CBD and antipsychotic use are each linked to hepatotoxicity and elevations in transaminase, which could be exacerbated when the two agents are co-administered (85). Drug metabolism barriers that limit the concomitant use of CBD and standard antipsychotic medications might be circumvented by optimizing the timing of when each agent is administered. To minimize competitive inhibition, CBD and antipsychotic medication administration should not overlap (94). Additionally, the CBD or antipsychotic medication dose could be decreased while monitoring for signs of adverse events including toxicity (85). The bioavailability of CBD can vary depending on the dosage form (85). Purified liquid forms that display more predictable bioavailability are recommended, with measurable drug levels and peak concentrations reached within 1-3 and 3-5 hours, respectively (85). Additional studies are required to fully determine optimal dosing parameters as well as overall safety and efficacy.

## Conclusion

Schizophrenia is a serious mental health disorder affecting 1% of adults worldwide (12, 30). Despite its low prevalence, it has one of the highest mortality rates (including significant premature mortality) amongst all psychiatric disorders (13). Many individuals with schizophrenia use illicit substances, especially cannabis (15). The two primary constituents of cannabis, delta-9-THC and CBD, exert opposite effects on schizophrenia (58, 59). Emerging data indicate that delta-9-THC exacerbates the positive symptoms associated with schizophrenia (58, 59). High-potency cannabis strains that reflect an increased concentration of delta-9-THC may elicit psychotic symptoms and cause cognitive deficits in individuals without schizophrenia (58). Moreover, illicit synthetic cannabinoids, distributed under the names K2, synthetic marijuana, and Spice, induce pronounced psychotomimetic symptoms in healthy individuals even at moderate doses (96). Contention about whether CBD significantly reduces schizophrenia-related psychosis remains, but some data suggest that it mitigates psychosis and could be used as an adjunct to current schizophrenia treatment regimens (58, 59). Namely, CBD alleviates positive symptoms of schizophrenia and ameliorates side effects associated with antipsychotic medications, potentially improving the poor adherence to treatment regimens in this patient population. As the potency of cannabis strains continues to rise, the increased delta-9-THC : CBD ratio could minimize the beneficial effects of CBD in schizophrenia and potentiate associated positive symptoms, although multiple lines of evidence indicate that CBD does not protect against the acute adverse effects of cannabis (97–99). Taken together, additional clinical studies assessing the safety and efficacy of incorporating CBD into schizophrenia treatment regimens as an adjunct therapy are necessary (100). If deemed safe and effective, CBD could potentially be incorporated into treatment guidelines. Given the over-the-counter availability as well as widespread notoriety and acceptance amongst the general public of CBD, it is paramount to fully elucidate the therapeutic utility of CBD to treat schizophrenia.

## Author contributions

KJ: Conceptualization, Investigation, Writing – original draft, Writing – review & editing. AW: Investigation, Writing – original draft, Writing – review & editing, Conceptualization. MB: Conceptualization, Investigation, Project administration, Supervision, Writing – original draft, Writing – review & editing.
